# Insulin-like growth factor 1 associated with altered immune responses in preterm infants and pigs

**DOI:** 10.1038/s41390-023-02794-w

**Published:** 2023-08-30

**Authors:** Ole Bæk, Martin Bo Rasmussen, Therese Gerts, Lise Aunsholt, Gitte Zachariassen, Per Sangild, Duc Ninh Nguyen

**Affiliations:** 1 Section for Comparative Pediatrics and Nutrition, Department of Veterinary and Animal Sciences, University of Copenhagen, Copenhagen, Denmark; 2 Copenhagen University Hospital, Rigshospitalet, Copenhagen, Denmark; 3 Hans Christian Andersen Children’s Hospital, Odense University Hospital, Odense, Denmark; 4 University of Southern Denmark, Odense, Denmark; 5 Open Patient data Explorative network, Region of Southern Denmark, Odense, Denmark

## Abstract

**Background:**

Preterm infants show low blood levels of insulin-like growth factor 1 (IGF-1), known to be negatively correlated with Interleukin-6 (IL-6). We hypothesized that circulating IGF-1 is associated with systemic immune-markers following preterm birth and that exogenous IGF-1 supplementation modulates immune development in preterm pigs, used as model for preterm infants.

**Methods:**

Plasma levels of IGF-1 and 29 inflammatory markers were measured in very preterm infants (*n* = 221). In preterm pigs, systemic immune development, assessed by in vitro challenge, was compared between IGF-1 treated (2.25 mg/kg/day) and control animals.

**Results:**

Preterm infants with lowest gestational age and birth weight showed the lowest IGF-1 levels, which were correlated not only with IL-6, but a range of immune-markers. IGF-1 supplementation to preterm pigs reduced plasma IL-10 and Interferon-γ (IFN-γ), IL-2 responses to challenge and reduced expression of genes related to Th1 polarization. In vitro addition of IGF-1 (100 ng/mL) further reduced the IL-2 and IFN-γ responses but increased IL-10 response.

**Conclusions:**

In preterm infants, plasma IGF-1 correlated with several immune markers, while supplementing IGF-1 to preterm pigs tended to reduce Th1 immune responses. Future studies should document whether IGF-1 supplementation to preterm infants affects immune development and sensitivity to infection.

**Impact:**

- Supplementation of insulin-like growth factor 1 (IGF-1) to preterm infants has been proposed to promote postnatal growth, but its impact on the developing immune system is largely unknown.
- In a cohort of very preterm infants, low gestational age and birth weight were the primary predictors of low plasma levels of IGF-1, which in turn were associated with plasma immune markers. Meanwhile, in immature preterm pigs, experimental supplementation of IGF-1 reduced Th1-related immune responses in early life.
- Supplementation of IGF-1 to preterm infants may affect the developing immune system, which needs consideration when evaluating overall impact on neonatal health.

## Introduction

Preterm infants, born before 37 weeks of gestation, are often hospitalized after birth and highly susceptible to infections, leading to a high incidence of sepsis and death.^1–3^ Factors contributing to this increased burden of infectious diseases include high-risk hospital procedures like catheterization, surgeries, and mechanical ventilation.^4,5^ In addition, the immune system of newborn preterm infants is also more immature, relative to that in term-born infants, further increasing their risk of infections.^6–9^ Insulin-like growth factor-1 (IGF-1) is an anabolic growth factor, important for many aspects of fetal and postnatal organ development,^10^ but its effects on systemic immune development are poorly investigated. Circulating IGF-1 binds to IGF-binding protein 3 (IGFBP-3) and, when unbound, activates the IGF-1 and insulin receptors to promote glucose uptake, cell proliferation, differentiation, and apoptosis inhibition.^11,12^ Insulin and IGF-1 receptors are co-expressed on almost all cell types, opening the possibility that IGF-1 affects the proliferation, maturation and function of the developing leucocytes.^12^ IGF-1 is also important for tissue repair mechanisms, promoting the production of interleukin-10 (IL-10) in T-cells and differentiation of anti-inflammatory M2 macrophages,^13–15^ indicating it may play a role in shaping the developing immune responses in infancy. The immune system of the fetus *in utero* is skewed towards tolerogenic Th2 responses to avoid potentially harmful immune reactions that could lead to fetal rejection and death.^16,17^ After birth, the neonatal immune system gradually shifts towards a Th1 phenotype to combat invading pathogens,^18^ a process that is delayed in preterm infants,^19,20^ leading to compromised immunity against viruses and intracellular pathogens.^21^

*In utero* and late gestation, IGF-1 is mainly supplied to the fetus via the placenta, before endogenous IGF-1 production by a variety of organs increases after birth. Preterm birth is therefore associated with a period of low IGF-1 levels until increasing endogenous production, mainly from the neonatal liver, secures higher circulating physiological levels.^10,22^ Low postnatal IGF-1 levels associate with postnatal growth restriction, as well as morbidities such as bronchopulmonary dysplasia (BPD), necrotizing enterocolitis (NEC), and retinopathy of prematurity (ROP).^10,23–26^ Thus, clinical trials have explored IGF-1 supplementation in preterm infants to prevent these disorders.^27–29^ Local and systemic inflammation is associated with BPD, NEC, and ROP, but whether IGF-1 directly or indirectly affects disease sensitivity via the developing immune system is unknown. There are indications that IGF-1 production is suppressed by interleukin 6 (IL-6) in preterm infants^30,31^ and that IL-6 may degrade IGFBP-3 thereby reducing plasma IGF-1 levels.^32^ However, investigations into a wider range of inflammatory mediators are lacking.

To better understand how IGF-1 affects systemic immune development following preterm birth, we first investigated circulating IGF-1 levels, together with a range of cytokines and chemokines in hospitalized very preterm infants. Next, preterm pigs were used as models for hospitalized preterm infants to investigate if supplemental IGF-1 would affect systemic immune development in the first three weeks of life. This period is characterized by rapid development of immune cell populations, which could impact susceptibility to infections and sepsis later in life. Preterm pigs, delivered at 90% gestation, are well established models for moderately to very preterm infants, showing clear signs of respiratory deficits, growth, and metabolic complications, impaired gut and brain development,^33,34^ together with depressed pro-inflammatory responses, high susceptibility to neonatal sepsis, low leucocyte counts and delayed postnatal Th1 polarization.^34–37^ These pigs also demonstrate low plasma IGF-1 levels, and supplementation with IGF-1 has shown improved gut resistance to NEC.^38^ However, the effects of such treatment on systemic immunity and other organ systems remain unclear.

## Methods

### Plasma levels of IGF-1 and inflammatory markers in very preterm infants

Data were used and extracted from a cohort of preterm infants (*n* = 221), as a part of a randomized controlled trial investigating effects of an alternative nutrient fortifier for very preterm infants (26–32 weeks of gestation, *FortiColos* trial, investigating efficacy of bovine colostrum as a nutrient fortifier, clinicaltrials.gov registration: NCT03537365, study protocol and main results published elsewhere^39,40^). Information on birth mode and pre/perinatal morbidities was recorded and infants were classified into small- or appropriate-for-gestational age (SGA, AGA) by birthweight according to the Fenton growth chart.^41^ Likewise, infants were classified as either very (29–32 weeks) or extremely (<29 weeks) premature. Blood samples were collected by capillary puncture (<1 mL, EDTA coated tubes) at three time points: Before, 1 and 2 weeks after the start of diet intervention, equivalent to approximately postnatal week 1, 2 and 3 weeks after birth. Samples were kept on ice until centrifugation for plasma collection, which was then frozen (−80 C) until later analyses. The main results of the trial are published elsewhere; in short, there was no significant effects of the diet intervention on growth indices (body weight, length, head circumference) or incidence of NEC, ROP or BPD, although bowel habits were improved.^40,42^ The intervention likewise did not influence circulating IGF-1 levels and had minor, transitory effects on interleukins 10 and 15 (unpublished data).

**Fig. 1:**
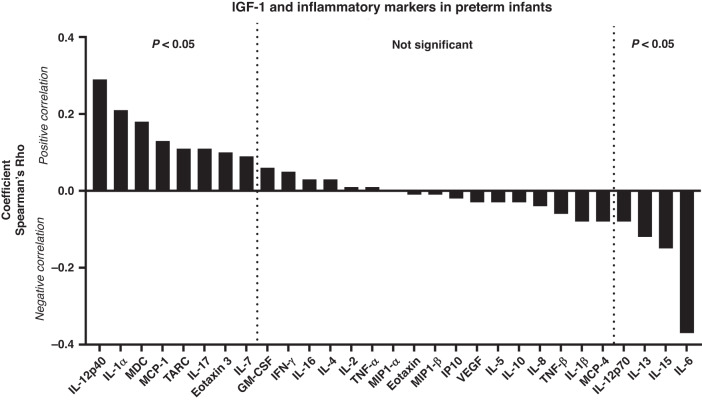
Correlation between plasma IGF-1 levels and plasma cytokine levels in very preterm infants during the first three weeks of life. Shown as Spearman’s Rho’s for each cytokines relation to IGF-1 levels. IL Interleukin, GM-CSF Granulocyte-macrophage colony-stimulating factor, IFN Interferon, IP10 Interferon gamma-induced protein 10, MCP Monocyte chemoattractant protein, MDC Macrophage-derived chemokine, MIP Macrophage inflammatory protein, TARC Thymus and activation regulated chemokine, TNF Tumor necrosis factor, VEGF Vascular endothelial growth factor.

Levels of IGF-1 were determined by an enzyme-linked immune assay, using 1:2 diluted samples as per the manufacturer’s instructions (E-20, Mediagnost, Reutlingen, Germany). In parallel, a series of cytokines and chemokines (total 29) were measured by three different fluorescent multiplex assays, as per the manufacturer’s instructions, using 1:4 diluted samples (V-plex chemokine, cytokine, and pro-inflammatory assays, respectively, Mesoscale, Rockville, Maryland). Levels of samples below the detection limit were set at 50% of the lowest standard of the assay. To explore potential associations between circulating IGF-1 and immune development, we performed non-parametric correlation analyses between IGF-1 levels and the levels of individual cytokines/chemokines in each blood sample obtained. All investigated cytokines are listed in Fig. 1.

### Supplementation with IGF-1 to newborn preterm pigs

#### Study design, birth, and nutrition

Forty-three preterm pigs (*Sus scrofa*, [Landrace x Duroc x Yorkshire]) from two litters were delivered by caesarian section at 90% gestation (day 106), quickly resuscitated with 100% oxygen, and if needed, treated with continuous positive airway pressure before being transferred to individual heated incubators. While still under anesthesia from the caesarian section, all pigs were fitted with an orogastric tube and an umbilical arterial catheter, enabling enteral and parenteral feeding as well as arterial blood sampling. Prematurely born piglets are highly susceptible to gut inflammation, potentially developing into NEC.^43,44^ As such, special feeding and rearing protocols are needed to improve long-term survival. Within the first 24 h, the pigs were supplemented with oral and parenteral administration of maternal plasma (25 mL/kg) to provide passive immunity. All animals were fed increasing amounts of raw bovine milk, fortified with whey protein (DI-9025, Arla foods ingredients, Viby, Denmark). Animals were also nourished by parenteral nutrition (PN, Kabiven, Fresenius Kabi, Copenhagen, Denmark) from birth until postnatal day 8, when umbilical catheters were removed. To prevent diarrhea and improve chances of survival, all animals were treated with oral antibiotics from day 8 to 10 (a combination of amoxicillin with clavulanic acid, metronidazole, and gentamicin). On day 10, animals were transferred to larger cages with free access to drinking water and in-cage environmental enrichments (toys) until sacrifice at day 19 of life. Full tissue collection was performed on all animals that survived until day 17. Animals euthanized before this time were subjected to necropsy to determine cause of death. A more detailed description of the preterm pig experimental setup and feeding/rearing procedures has been published elsewhere.^43^

#### IGF-1 supplementation

After birth, animals were stratified by birth weight and sex and randomly allocated to either IGF-1 (*n* = 21) or control treatment (*n* = 22). Recombinant human (rh)IGF-1/rhIGFBP-3 complex (Mecasermin rinfabate; Takeda pharmaceuticals, Cambridge, Massachusetts, hereafter denoted IGF-1) was first supplied continuously from day one until eight by mixing it into the PN solution (2.25 mg/kg/day). Doses were based on previous animal and preterm infant pilot trials.^27–29,38,45^ From days 9–19, the IGF-1 treatment was given subcutaneously, 0.75 mg/kg every 8 h using a subcutaneous catheter, which was replaced every three days. Control animals were given the same volumes of vehicle solution in the same manner as their IGF-1 treated counterparts. All personnel handling the animals were blinded to the treatments.

#### Blood sampling and evaluation of immune function

Blood was collected on day 8 via the arterial catheter (before antibiotic treatment) and on day 19 by jugular vein puncture. Blood was either transferred to EDTA- or heparin-containing tubes and centrifuged to yield plasma for further immune analyses. Hematological parameters in whole blood were assessed by an Advia 2120 hematology system (Siemens Healthcare Diagnostics, Malvern, Pennsylvania). Neutrophil phagocytic function was evaluated by incubating whole blood with fluorescently labeled *E. coli* (pHrodo, Thermo Fisher Scientific, incubated 30 min at 37 °C), after which the samples were analyzed by a flow cytometer (Accuri C6, BD Biosciences, Franklin Lakes, New Jersey), as described in detail elsewhere.^35^ Neutrophil phagocytic rate was defined as the fraction of neutrophils with internalized bacteria and phagocytic capacity as the median fluorescent index of neutrophils with internalized bacteria.

To evaluate the immune function, we performed in vitro whole blood stimulation assays, using fresh blood collected on days 8 and 19. Whole blood samples were incubated (37 °C) with either live *Staphylococcus epidermidis* (SE, WT-1457, 2*10^6^ CFU/mL, 2 h), phorbol 12-myristate 13-acetate/ionomycin (PMA, 25 ng/mL + 1 µg/mL, 5 h) or appropriate controls (phosphate buffered saline or dimethyl sulfoxide, respectively). In the in vitro assays, the SE or PMA stimulated samples were additionally treated with either IGF-1 (100 ng/mL) or formulation buffer. After incubation whole blood RNA was preserved (MagMax lysis buffer, Thermo Fisher Scientific, Allerød, Denmark) for later analysis of leucocyte gene expression (kept at −80 °C) and levels of tumor necrosis factor alpha, interferon gamma, and interleukins 2 and 10 (TNF-α, IFN-γ, IL-2, IL-10) in assay supernatants were measured by enzyme-linked immunoassays, using porcine-specific antibodies (Dousets, R&D systems, Minneapolis, Minnesota). Results below the detection limit were set to 50% of the lower detection limit of the assay.

Leucocyte gene expression analysis was performed only in whole blood stimulated with SE or appropriate control. A detailed description of the procedures has been published elsewhere^37,46^ Briefly, RNA was extracted from preserved blood from the in vitro assays (MagMax 96 blood RNA isolation kit, Thermo Fisher Scientific) and RNA contents measured by spectroscopy (Nanodrop 1000, Thermo Fisher Scientific). Using quantitative polymerase chain reaction (qPCR, using QuantiTect SYBR Green PCR Kit, Qiagen, Venlo, Netherlands) on a LightCycler 480 system (Roche, Basel, Switzerland), we determined the expression of 23 genes related to inflammation and metabolism. All gene expressions were shown relative to *HPRT1* expression as a reference. A full list of primers and genes investigated is shown in Supplementary Table S1.

### Statistics

All statistics were performed using Stata (v. 14.2, Stata Corp., College Station, Texas). Differences in IGF-1 levels in the preterm infants were calculated by students *T*-test and increases in IGF-1 over the first three weeks of life were calculated by a linear mixed model with postnatal time, as fixed effects and ID as a random effect. Correlations between cytokine and IGF-1 levels in preterm infants were analyzed using Spearman’s rank correlation test. Correlations between each cytokine/chemokine and IGF-1 were done independently within each blood sample.

Data from the preterm pig study were analyzed with a linear mixed effect model using IGF-1 treatment, sex and birth weight as fixed factors, and litter as the random factor. For repeated measures, pig ID was also used as a random factor. If necessary, data were logarithmically transformed to obtain normal distribution, and if no acceptable model could be fitted, a non-parametric test was performed (Mann–Whitney *U* test for direct comparisons and Wilcoxon’s test for paired samples).

## Results

### Correlations between circulating IGF-1 and inflammatory markers in very preterm infants

From the retrospective cohort 587 plasma samples from 221 infants (three samples from most infants) were collected at different time points during hospitalization. The earliest sample for each infant was collected at 7.5 days of life (week 1, with a standard deviation of ±1.6 days). The two consecutive samples were collected at 14.2 days (±1.8 days, week 2) and 21.4 days (±1.7 days, week 3) of life. Early in life, IGF-1 levels were lower in infants born before 29 weeks of gestation and SGA infants (Table 1, *P* < 0.001 and < 0.01, respectively), and tended to be lower in infants born following preeclampsia (Table 1, *P* = 0.08). Over the first 3 weeks of life, IGF-1 levels gradually increased in all groups, except in SGA infants and those born before week 29 of gestation, where IGF-1 levels remained lower than in their higher weight and gestational age counterparts (Table 1).

**Table 1:** Plasma IGF-1 levels in very preterm infants over the first 3 weeks of life.

	*n*	IGF-1 levels (ng/mL)^a^			*P* time
		Week 1	Week 2	Week 3	
Sex					
Male	125 (57%)	27.4 (9.9)	28.8 (13.3)	32.0 (14.7)	<0.05
Female	96 (43%)	28.7 (11.8)	33.2 (16.1)	34.8 (15.3)	<0.01
Birthweight					
AGA	172 (78%)	29.2 (11.2)	33.6 (15.6)	34.8 (14.5)	<0.001
SGA	49 (22%)	24.0 (3.4)**	23.4 (8.5)***	28.0 (15.4)**	NS
Preeclampsia					
No	168 (76%)	28.7 (11.2)	31.4 (15.5)	33.7 (14.6)	<0.001
Yes	52 (24%)	25.9 (8.9)*	28.2 (11.5)	31.7 (16.5)	<0.05
Caesarian section					
No	62 (28%)	26.9 (8.2)	31.4 (14.0)	33.4 (13.8)	<0.05
Yes	159 (72%)	28.6 (11.7)	30.4 (14.9)	33.2 (15.5)	<0.001
Premature rupture of membranes					
No	172 (78%)	27.9 (11.1)	31.1 (15.0)	32.9 (15.0)	<0.001
Yes	49 (22%)	28.7 (10.2)	29.0 (13.0)	34.6 (15.0)	<0.05
Antenatal steroids					
No	10 (5%)	26.9 (8.6)	34.6 (18.6)	32.4 (11.6)	<0.05
Yes	211 (95%)	28.2 (11.0)	30.5 (15.5)	33.3 (15.2)	<0.001
Gestational age					
29–32 weeks	106 (48%)	31.9 (11.0)	37.3 (15.8)	40.8 (16.1)	<0.001
<29 weeks	115 (52%)	24.5 (9.4)***	23.6 (9.2)***	26.9 (10.4)***	NS

Data are shown as means with corresponding standard deviations.

*AGA* Appropriate for gestational age, *SGA* Small for gestational age, *NS* Not significant.

*Difference between groups, *P < 0.1, **P < 0.01, ***P < 0.001.

^a^Plasma IGF-1 levels in samples collected 1-3 weeks after birth.

Spearman’s correlation analyses between plasma IGF-1 and inflammatory markers across the three time points are shown in Fig. 1, ranked from most positive to negative correlation. The markers most positively associated with IGF-1 levels (e.g., p40 subunit of IL-12 [IL-12p40], IL-1α, macrophage-derived chemokine [MDC], macrophage chemoattractant protein 1 [MCP-18] and thymus and activation regulated chemokine [TARC] and IL-7) can generally be considered involved in Th2 polarization, thymus development and/or macrophage/monocyte activation. Conversely, inflammatory markers negatively associated with IGF-1 levels (e.g., p70 subunit of IL-12 [IL12p70] and IL-15) can be considered related to Th1 polarization. Exceptions to this general picture were the Th2 polarizing cytokines, IL-13 and IL-6, showing clear negative correlations with IGF-1 levels.

### IGF-1 supplementation and immune development in preterm pigs

#### Clinical effects of IGF-1 treatment

Birth weights of the preterm pigs were similar between groups and body growth was not affected by IGF-1 supplementation (data not shown). Of the 43 randomized animals, 29 survived until postnatal day 19 (14 control, 17 IGF-1) where levels of IGF-1 in plasma were three times higher in supplemented versus placebo animals (139 vs 43 ng/mL, *P* < 0.001). Mortality was primarily associated with bowel obstruction or necrotizing enterocolitis. Below, we report the effects on systemic immune parameters. Results related to metabolism, gut structure/function, survival and other organs will be reported elsewhere (unpublished).

#### Minor impact of IGF-1 on circulating leucocyte levels and neutrophil function

**Fig. 2:**
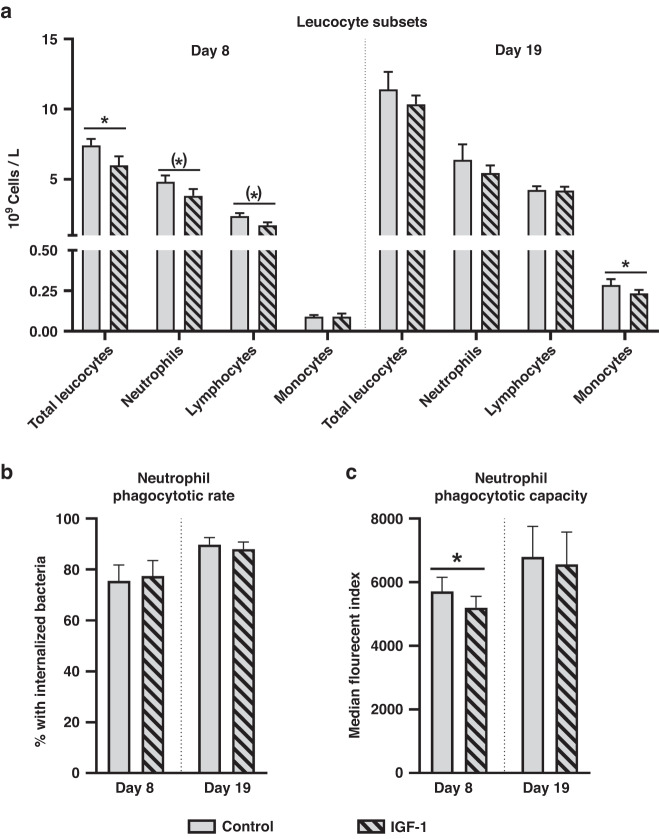
Leucocyte subsets and neutrophil function. Leucocyte counts in IGF-1 supplemented and control preterm pigs on days 8 and 19 (**a**) along with evaluation of neutrophil phagocytic rate (**b**) and capacity (**c**). Data presented as means with corresponding standard error. *Difference between groups. (*)*P* < 0.1, *P < 0.05.

On day 8 of life, IGF-1 supplemented preterm pigs showed lower levels of total leucocytes, mainly explained by lower neutrophil and lymphocyte levels (Fig. 2a). However, by day 19 total leucocyte levels, levels and fractions of leucocyte subsets were similar between the two groups, except that monocyte levels were lower in IGF-1 supplemented pigs (Fig. 2a). Other hematological parameters did not differ between groups and is shown in Supplementary Table S2. We further investigated neutrophil development by counting the number of circulating banded neutrophils, which were numerically elevated in IGF-1 supplemented animals, although not significantly (Supplementary Table S2). The overall neutrophil phagocytic rate did not differ between treatments (Fig. 2b), but the phagocytic capacity was lower in IGF-1 supplemented animals on day 8 (Fig. 2c). At euthanasia the relative weight of the spleen was highest in IGF-1 supplemented animals (4.2 ± 0.3 vs 2.8 ± 0.2 g/kg of body weight, *P* < 0.01).

#### IGF-1 affects whole blood cytokine production in vitro

**Fig. 3:**
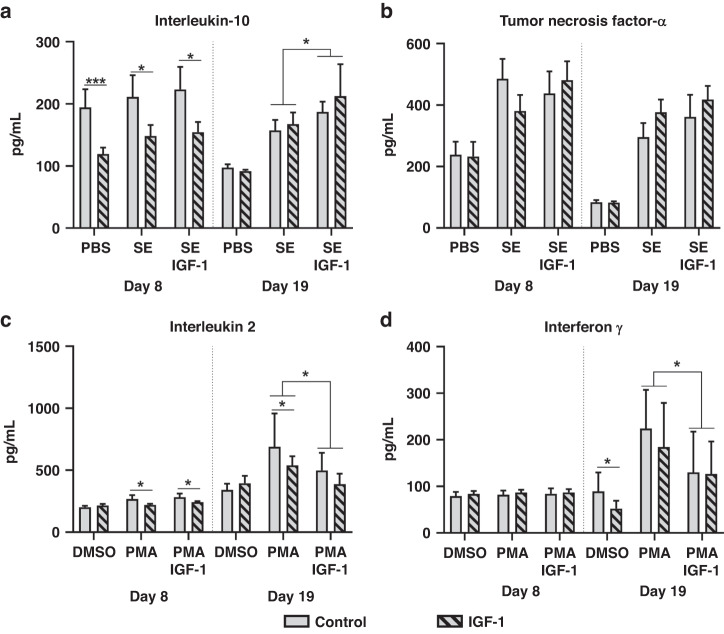
Effects of in vivo and in vitro IGF-1 supplementation on cytokine production in stimulated-whole blood. Levels of tumor necrosis factor α and interleukin 10, following stimulation with live *Staphylococcus epidermidis* or phosphate buffered saline control (SE/PBS, **a**, **b**) as well as interleukin 2 and interferon γ, following stimulation with phorbol 12-myristate 13-acetate/ionomycin or Dimethylsulfoxide control (PMA/DMSO, **c**, **d**). Data are shown for IGF-1 and control animals, with or without further in vitro addition of IGF-1 (100 ng/ml). Data are presented as means with corresponding standard error. Significant differences between groups are indicated, *P < 0.05, **P < 0.01 and ***P < 0.001.

To evaluate the immune competence, whole blood was subjected to in vitro stimulation with either SE or PMA and production of key cytokines was measured. To further evaluate the direct effects of IGF-1 on immune cells, samples were treated with additional IGF-1 (100 ng/ml) or appropriate control. IGF-1 supplemented preterm animals showed lower cytokine levels of IL-10 on day 8, both in the stimulated and unstimulated samples (Fig. 3). By day 19, group differences were no longer observed, and both IGF-1 and placebo treated animals mounted similar IL-10 responses. In vitro addition of IGF-1 to whole blood further increased IL-10 production for both groups (Fig. 3a). In contrast, no effects of IGF-1, either in vivo or in vitro, were observed on TNF-α basal levels or responses to challenge (Fig. 3b).

IGF-1 treated animals were not able to mount IL-2 responses; however, further in vitro addition of IGF-1 on day 19 reduced IL-2 responses in both IGF-1 and control pigs (Fig. 3c). For IFN-γ, no effects of IGF-1 treatment were observed on day 8 (limited response upon stimulation, Fig. 3d). By day 19, basal levels of IFN-γ were lower in the IGF-1 treated animals, while further in vitro addition of IGF-1 lowered the levels of this cytokine in both groups (Fig. 3d). Combined, PMA stimulation tests indicated that blood leucocytes were skewed less to Th1 polarization in IGF-1 versus control animals.

#### IGF-1 supplementation affects whole blood leucocyte gene expressions

**Fig. 4:**
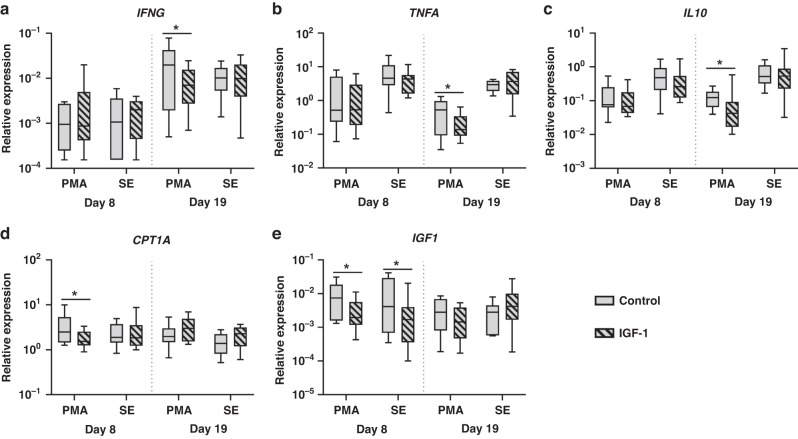
Effects of in vivo IGF-1 supplementation on whole blood gene expressions. Expression of genes related to adaptive immune responses (IFNG, TNFA, IL10, **a**–**c**), fatty acid metabolism (CPT1A, **d**) and endogenous IGF1 (**e**), in whole blood; either unstimulated (PBS) or stimulated with Staphylococcus epidermidis (SE), on day 8 and 19 in preterm pigs supplemented with either IGF-1 or control from birth. Data are presented as relative gene expressions, using range plots. *Difference between groups, *P < 0.05.

**Fig. 5:**
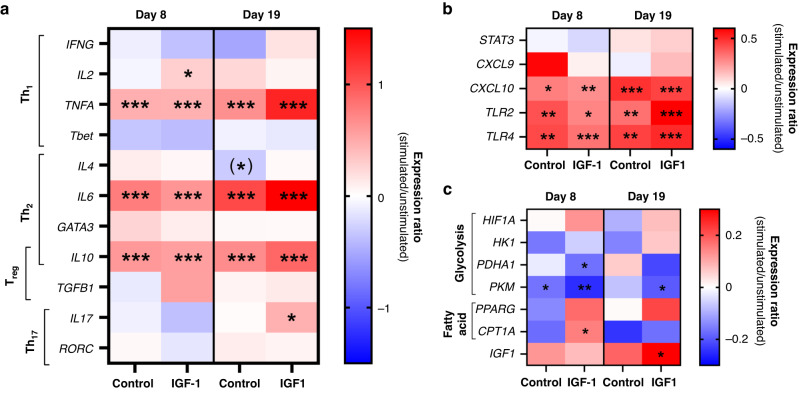
Changes in whole blood gene expression over time following in vivo IGF-1 supplementation. Effect of stimulation with *Staphylococcus epidermidis* (SE) on the expression of genes related to T cell polarization (**a**), innate immunity (**b**) and cellular metabolism (**c**). The heat maps show the ratio of gene expression (log scale) in stimulated versus unstimulated samples on days 8 and 19 in preterm pigs supplemented with IGF-1 or control. *Effect of stimulation with SE, *P < 0.05, **P < 0.01 and ***P < 0.001.

We examined the expression of genes related to immunity and metabolism (Fig. 4) and their response to SE stimulation (Fig. 5). For genes related to T cell polarization, no effects of IGF-1 were apparent on day 8, but the basal expression of *IFNG, TNFA* and *IL10* on day 19 was lower in IGF-1 treated animals (Fig. 4a–c), suggesting a less Th1 polarized immune status in these animals. None of the genes related to innate immunity (*TLR2, TLR4, CXCL9, CXCL10*) were affected by IGF-1 treatment. Among energy metabolism-related genes, only *CPT1A* level was higher in IGF-1 treated animals on day 8 (Fig. 4d). Genes related to glycolysis were not affected by IGF-1 treatment (data not shown), indicating that IGF-1 may enhance cellular fatty acid oxidation. Endogenous blood cell *IGF1* gene expression was lower in IGF-1 supplemented animals on day 8 (both unstimulated and stimulated samples) but this effect disappeared by day 19 (Fig. 4f).

Upon stimulation with SE, only IGF-1 treated animals showed upregulated *IL2* and *IL17* expression (on days 8 and 19, respectively, Fig. 5a), indicating some reduced baseline expression of these genes. Likewise on day 19, only control, not IGF-1 supplemented animals, tended to have lower *IL4* expression in response to SE stimulation (Fig. 5a, *P* = 0.06). Both groups showed strong elevation of innate immune genes (*TLR2, TLR4* and *CXCL10*) in SE stimulated vs. unstimulated samples (Fig. 5b). Importantly, several energy metabolism-related genes showed SE stimulation effects only in IGF-1 animals, including down-regulation of glycolysis-related gene *PDHA1* on day 8 and *PKM* on day 19, as well as up-regulation of *CPT1A* on day 8. Again, this indicates that IGF-1 may affect leucocyte energy metabolism to favor fatty acid metabolism, potentially enhancing anti-inflammatory effects, relative to glycolysis-enhancing pro-inflammatory effects and Th1 polarization. Finally, only IGF-1 supplemented animals were able to upregulate their blood IGF-1 expression upon SE stimulation on day 19 (Fig. 5c).

## Discussion

In various studies involving preterm infants, higher circulating levels of IGF-1 have been linked to improved growth and health outcomes.^23,26^ However, it remains challenging to determine whether these associations indicate causal effects or indirect relationships. In addition, circulating IGF-1 levels, mainly produced by the liver, poorly reflects local synthesis and actions of the growth factor across multiple tissues with a widely different distribution of IGF-1 and insulin receptors. Preterm infants often show a period of very low circulating IGF-1 levels and small-scale clinical studies have tested the effects of low-dose physiologic IGF-1 supplementation with inconclusive effects on ROP, IVH, and NEC outcomes, but with consistent preventative effects on BPD.^27,28,45^ As many diseases of prematurity associate with systemic immune dysfunctions and inflammation, it is critical to better understand how circulating IGF-1 levels may or may not be directly related to immune development.

In our cohort of preterm infants, we generally observed low levels of circulating IGF-1 compared to term infants.^10^ Our findings align with previous studies indicating that low gestational age and birth weight are the primary factors contributing to diminished IGF-1 levels.^22,26^ In this perspective, preterm infants with the lowest gestational age (e.g., extremely preterm infants, <29 weeks GA), born with or without SGA and/or after preeclampsia, may benefit most from raising circulating IGF-1 levels in the weeks after birth. Among the cytokines and chemokines examined, those exhibiting a positive correlation with IGF-1 in preterm infants were primarily associated with Th2-polarized immune responses, thymus development, and/or macrophage activation (IL-12p40, MDC, MCP1, TARC, IL-7). This is in line with in vitro studies have shown that co-stimulation with IGF-1 promotes an M2 phenotype in adult macrophages.^14^ In contrast, immune factors with negative relationship to IGF-1 were involved in Th1 or IL-6 responses (IL-6, IL-13, IL12p70, IL-15). However, it is important to note that the correlations between cytokine and IGF-1 levels, although significant, were quite low. As such IGF-1 does not seem a major predictor of cytokine levels, yet the results confirm the known negative correlation between IGF-1 and IL-6 levels in preterm infants.^30,31^ Previous animal and in vitro studies show that IL-6 may directly inhibit the synthesis of IGF-1 and initiate breakdown of IGF1BP-3.^32,47^ Elevated IL-6, together with low IGF-1 levels, are also found in children and adults suffering from growth failure or infection with human immunodeficiency virus.^10,31,48^ Likewise, in preterm infants, IL-6 and IGF-1 levels in cord blood are inversely correlated,^30^ while preterm infants with circulating IGF-1 levels <20 ng/mL showed elevated IL-6 levels.^31^

To further investigate if circulating IGF-1 levels directly induce systemic immunomodulatory effects in a state of prematurity, we conducted a preterm pig study on IGF-1 supplementation. In contrast to rodents, preterm pigs allow assessment of the immaturities of systemic immune responses associated with low GA at birth, together with low IGF-1 levels and a range of morbidities known from preterm infants.^34,35,49,50^ In the current study, systemic supplementation of IGF-1 led to few, but consistent effects on the immune ontogeny. In the first week of life, preterm pigs are severely neutropenic and show poor immune responses to pathogen challenge.^35,49^ In a previous study, IGF-1 supplementation to similarly raised preterm pigs increased neutrophil counts on day 5 of life.^38^ It has previously been speculated that IGF-1 exerts trophic effects on the bone marrow and may lead to faster expansion of the leucocyte pool,^12^ but in contrast, we observed what appeared as an IGF-1 related delay in hematopoiesis. However, the relative number of immature neutrophils may have increased, leading to the observed negative effects on neutrophil phagocytic function in IGF-1 treated animals. Nonetheless, spleen size was markedly increased (almost +100%), an observation we have previously shown also in 5–9 day-old preterm pigs supplemented with IGF-1.^38^ Previous studies on adult mice have revealed similar effects of IGF-1 on spleen size, driven by proliferation of B and T cells.^51^ IGF-1 may therefore have proliferative effects on immune cells in primary and secondary lymphoid organs. This hypothesis clearly warrants further investigation.

Although the overall hematological effects were marginal, IGF-1 supplementation affected baseline cytokine levels and responses to in vitro stimulation in an age-dependent manner. IGF-1 supplemented pigs showed lower levels of IL-10 (only on day 8) and IFN-γ (only on day 19), while TNF-α and IL-2 levels were similar between groups. Lower levels of IL-10 on day 8 could be influenced by the differences in circulating immune cells or differences in overall inflammatory states. Gene expression data also indicated time-dependent effects, with minimal IGF-1 effects observed on day 19, resulting in reduced baseline expression of *IFNG* and *TNFA*. Cellular energy metabolism-related gene expression data suggested that IGF-1 supplementation favored fatty acid/mitochondrial oxidative phosphorylation over glycolysis in immune cells stimulated with live bacteria, opening the possibility that IGF-1 can affect the function of immune cells by affecting their energy metabolism. This also supports the idea that IGF-1 supplementation promotes Th2 immunity, as pro-inflammatory Th1 responses typically rely on glycolysis for rapid energy generation.^52^ Possibly this effect was only temporary as the overall effects of IGF-1 on cytokine production were most pronounced on day 8, just after a major expansion of immune cell populations in preterm pigs^35^, relative to day 19. In vitro addition of IGF-1 directly to the whole blood assay confirmed these findings by dampening IL-2 and IFN-γ responses while enhancing the IL-10 response. Previous finding also show that *in vitro co-stimulation with* IGF-1 dampens the IFN-γ responses to PMA challenge in infant cord blood.^53^ The data suggest that IGF-1 may delay the normal postnatal transition towards a more Th1-skewed phenotype, potentially leading to reduced resistance against certain pathogenic infections. An IGF-1 induced reduction of IFN-γ, or other Th1 polarized immune responses in neonates could reduce the risk of hyper-inflammatory responses to both systemic and local infections. These effects may also be involved in the observed reduction in NEC incidence in IGF-1 supplemented preterm pigs^38^ and for other possible effects on other organ systems, such as eyes (ROP) and lungs (BPD) in preterm infants.^26,28^ However, it is difficult to separate any direct immunomodulatory effects on such organs from IGF-1 related effects on tissue perfusion, vascularization and organ maturation.^23^

It remains to be shown if any IGF-1 mediated delay in IFN-γ or other Th1 related responses, predisposes preterm neonates to a higher risk of intracellular bacterial and viral infections. Only one phase 2 clinical trial of IGF-1 supplementation to preterm infants over an extended period has been conducted (*n* = 121), showing that IGF-1 supplementation prevented the development of BPD^28^. However, there were more cases of sepsis in the IGF-1 treated group (38 vs. 25%), although the difference was statistically insignificant. Smaller pilot studies, which short follow-up periods, have revealed no increased short-term risk of bacterial infections related to IGF-1 supplementation^27,29^. Ongoing larger-scale studies in preterm infants, having lung development and BPD as main outcomes (ClinTrials registration NCT03253263) will help to clarify how IGF-1 supplementation may increase or decrease sensitivity to bacterial and viral infections. Because systemic inflammatory responses have wide implications for the development of diverse organs in preterm neonates, including the brain, such studies are obligatory before IGF-1 therapy can be recommended for preterm infants suffering from low postnatal IGF-1 levels. Further preterm pig studies, involving bacterial challenge studies and a wider range of investigations into internal organs, may help to answer these important questions for the safety and efficacy of IGF-1 therapy.

In conclusion, our study suggests that IGF-1 can modulate the immune ontogeny of preterm neonates, via dampening Th1 and possibly promoting Th2 associated immune responses, although the effects were marginal. The immediate clinical impact of this association is unclear and IGF-1 therapy for preterm infants may potentially act as a double-edged sword; as reduced Th1 immunity may slow the development of hyperinflammatory disorders, but increase the risk of neonatal infections, especially with intracellular viral and bacterial pathogens. Possibly, IGF-1 supplementation is relevant to consider for subgroups of very/extremely preterm infants with additional complications such as being born after fetal growth restriction and inflammation, negatively affecting both IGF-1 production and immune development.

### Supplementary information

Supplementary tables

## Data Availability

The datasets generated during and/or analyzed during the current study are available from the corresponding author on reasonable request.
