# Supplementary material for: Insulin-like growth factor 1 associated with altered immune responses in preterm infants and pigs
Source: Pediatr Res. 2023 Aug 30;95(1):120–8. doi: 10.1038/s41390-023-02794-w (PMC10798898; doi:10.1038/s41390-023-02794-w)
Supplement: Supplementary file 1 — Supplementary tables [file 41390_2023_2794_MOESM1_ESM.pdf]

Supplementary Table S1: List of primer sequences used and genes investigated.

Gene	Protein	Forward sequence (5'-3')	Reverse sequence (5'-3')	Amplicon length*
HPRT1	Hypoxanthine Phosphoribosyl Transferase 1	TATGGACAGGACTGAACGGC	ACACAGAGGGCTACGATGTG	75
CPT1A	Carnitine Palmitoyl Transferase 1A	AAGATGGGCATGAACGCTGA	GTGGCCGTCCTCCTCATAAC	114
CXCL9	CXC chemokine ligand 9	GAAAAGCAGTGTTGCCTTGCT	TGATGCAGGAACAACGTCCAT	98
CXCL10	CXC chemokine ligand 10	ATCATCCCGAGCTGTTGAGC	CCAGGACTTGGCACATTCAC	94
GATA3	GATA binding protein 3	ACCCCTTATTAAGCCCAAGC	TCCAGAGAGTCGTCGTTGTG	92
HIF1A	Hypoxia-inducible factor 1 alpha	TGTGTTATCTGTCGCTTTGAGTC	TTTCGCTTTCTCTGAGCATTC	96
HK1	Hexokinase 1	TTTCCCTTGTCGGCAATCCA	CCTCCACTCCGCTTGCTTTA	80
IFNG	Interferon gamma	AGCTTTGCGTGACTTTGTGT	ATGCTCCTTTGAATGGCCTG	247
IL2	Interleukin 2	AAGCTCTGGAGGGAGTGCTA	CAACAGCAGTTACTGTCTCATCA	159
IL4	Interleukin 4	GTACCAGCAACTTCGTCCAC	CCTTCTCCGTCGTGTTCTCT	150
IL6	Interleukin 6	TGCCACCTCAGACAAAATGC	AGGTTCAGGTTGTTTTCTGCC	159
IL10	Interleukin 10	GTCCGACTCAACGAAGAAGG	GCCAGGAAGATCAGGCAATA	73
IL17	Interleukin 17	GCACACGGGCTGCATCAACG	TGCAACCAACAGTGACCCGCA	149
IGF1	Insulin-like growth factor 1	ATTTCTTGAAGGTAAAGATGCA	CAGCCCCACAGAGGGTCTCA	117
PDHA1	Pyruvate dehydrogenase α1	GTCAGGAAGCTTGTTGCGTG	GGTAAAGCCATGAGCTCGGT	86
PKM	Pyruvate kinase M1/2	GCCCTGGACACTAAAGGACC	CAGCCACAGGACATTCTCGT	147
PPARG	Peroxisome proliferator activated receptor gamma	TGACCATGGTTGACACCGAG	GATCAGCTCTCGGGAATGGG	128
RORC	Retinoic acid-related orphan receptor	CAGCGCTCCAACATCTTCTC	GACCAGCACCACTTCCATTG	207
STAT3	Signal Transducer and Activator of Transcription 3	ACATCCTTGTGTCTCCGCTG	GTATGGGGCAGCACTACCTG	125
T-bet	T-Box transcription factor	CTGAGAGTCGCGCTCAACAA	ACCCGGCCACAGTAAATGAC	121
TGFB1	Transforming growth factor beta 1	GCAAGGTCCTGGCTCTGTA	TAGTACACGATGGGCAGTGG	97
TLR2	Toll-like receptor 2	CGTGTGCTATGACGCTTTCG	GTACTTGCACCACTCGCTCT	232
TLR4	Toll-like receptor 4	TGGTGTCCCAGCACTTCATA	CAACTTCTGCAGGACGATGA	116
TNFA	Tumor necrosis factor alpha	ATTCAGGGATGTGTGGCCTG	CCAGATGTCCCAGGTTGCAT	120

*: Length in base pairs

Supplementary Table S2: Hematological parameters in preterm pigs supplemented with IGF-1

	Day	Placebo	IGF-1	P
Lymphocyte fraction (% of leucocytes)	8	33.1 (3.1)	30.9 (3.7)	NS
	19	41.5 (4.0)	41.8 (3.0)	NS
Monocytes fraction (% of leucocytes)	8	1.2 (0.2)	1.6 (0.4)	NS
	19	2.8 (0.3)	2.4 (0.3)	NS
Neutrophil fraction (% of leucocytes)	8	63.9 (3.0)	62.7 (3.2)	NS
	19	51.9 (4.1)	51.8 (2.6)	NS
Banded neutrophils (10^9 cells/L)	8	0.075 (0.05)	0.133 (0.07)	NS
	19	0.100 (0.07)	0.328 (0.31)	NS
Banded neutrophil fraction (% of neutrophils)	8	1.2 (0.6)	3.9 (2.0)	NS
	19	1.3 (0.8)	5.7 (5.4)	NS
Basophils (10^9 cells/L)	8	0.1 (0.0)	0.2 (0.1)	NS
	19	0.4 (0.2)	0.3 (0.1)	NS
Basophils fraction (% of leucocytes)	8	0.1 (0.0)	0.2 (0.1)	NS
	19	0.4 (0.2)	0.3 (0.1)	NS
Eosinophils (10^9 cells/L)	8	0.03 (0.01)	0.02 (0.0)	NS
	19	41.5 (4.0)	41.8 (3.0)	NS
Eosinophils fraction (% of leucocytes)	8	0.4 (0.1)	0.4 (0.1)	NS
	19	1.1 (0.3)	0.8 (0.2)	NS
Platelets (10^9cells/L)	8	427 (80)	363 (37)	NS
	19	609 (86)	714 (73)	NS

Data are shown as means with corresponding standard errors. NS: Not significant
